# Supplementary material for: An Evaluation of the Impact of a Multicomponent Stop Smoking Intervention in an Irish Prison
Source: Int J Environ Res Public Health. 2021 Nov 15;18(22):11981. doi: 10.3390/ijerph182211981 (PMC8624287; doi:10.3390/ijerph182211981)
Supplement: Supplementary file 1 [file ijerph-18-11981-s001.zip › ijerph-1431283-supplementary.pdf]

International Journal of
Environmental Research and Public Health

MDPI

Supplementary Materials

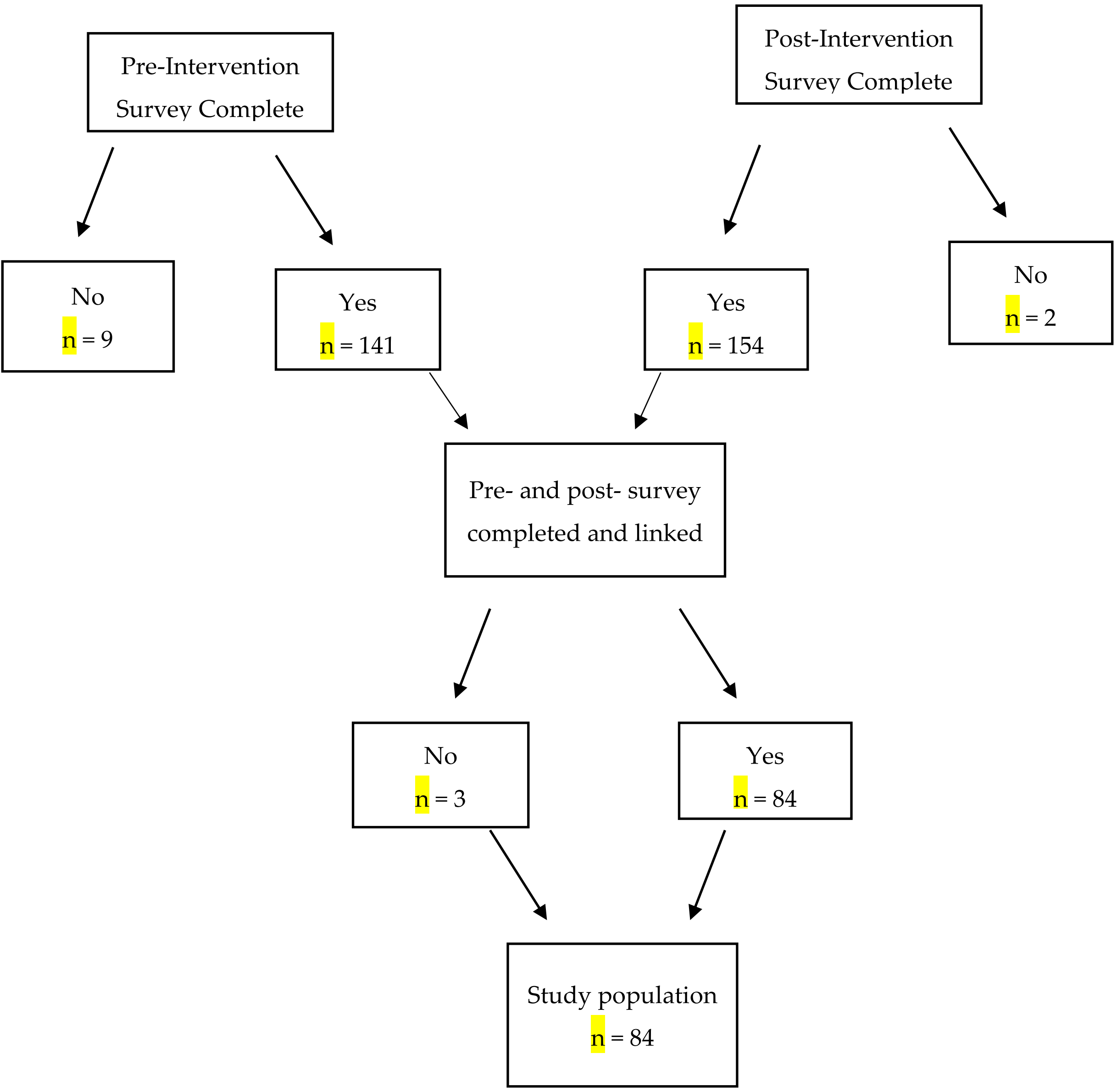

Figure S1. Flowchart of the Prisoner Study Population.

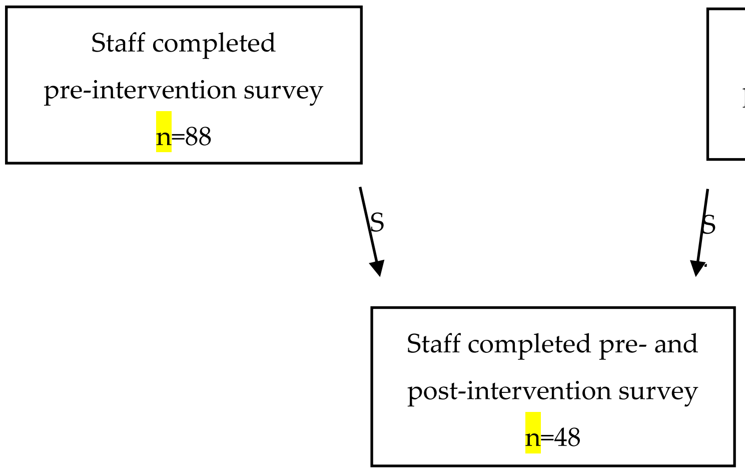

Figure S2. Flowchart of the Staff Study Population.

Table S3. Questionnaire Items related to smoking behaviours and dependency, health status, and exposure to second-hand smoke.

<u>**Smoking and quitting behaviours**</u>

1. **Do you smoke tobacco products?**

   Yes, daily ☐
   Yes, occasionally ☐
   No ☐

2. **Did you ever smoke tobacco products in the past?**

   Yes, daily ☐
   Yes, occasionally ☐
   No ☐

<u>**If you are a smoker, please answer the following questions**</u>

3. **How soon after you wake up do you smoke your first cigarette?**

   After 60 minutes ☐
   31-60 minutes ☐
   5-30 minutes ☐
   Within 5 minutes ☐

4. **Do you find it difficult to refrain from smoking in places where it is forbidden?**

   Yes ☐ No ☐

5. **Which cigarette would you hate most to give up?**

   The first in the morning ☐ Any other ☐

6. **How many cigarettes per day do you smoke?**

   10 or less ☐
   11-20 ☐
   21-30 ☐
   31 or more ☐

7. **Do you smoke more frequently during the first hours after awakening than during the rest of the day?**

   Yes ☐ **No** ☐

8. **Do you smoke even if you are so ill that you are in bed most of the day?**

Yes ☐ No ☐

Health status

9. **Would you say your health is:**

Excellent ☐

Very good ☐

Good ☐

Fair ☐

Poor ☐

10. **In the last 4 weeks:**

Have you had any whistling/wheezing in your chest? Yes ☐ No ☐

Have you felt short of breath? Yes ☐ No ☐

Do you usually cough first thing in the morning? Yes ☐ No ☐

Do you cough at all during the rest of the day? Yes ☐ No ☐

Do you bring up phlegm? Yes ☐ No ☐

11. **In the last 4 weeks:**

Have your eyes been red or irritated? Yes ☐ No ☐

Have you had a runny nose, sneezing or nose irritation? Yes ☐ No ☐

Have you had a sore or scratchy throat? Yes ☐ No ☐

12. **Please record the CO level as measured here ____________**

Environmental exposure to tobacco smoke/second-hand smoke

13. **Are you exposed to other people's cigarette smoke in the progression unit in Mountjoy prison?**

Yes ☐ No ☐

14. **During the past 7 days, how many hours were you exposed to other people's cigarette smoke?**

Never or almost never ☐

Less than 1 hour per day ☐

1-5 hours per day 6- ☐

6-10 hours per day ☐

10 hours or more per day ☐

Table S4. Total Baseline Participant Characteristics.

.	Total Baseline Population [n = 229]		Baseline Prisoner Population [n=141]		Prisoner Longitudinal Cohort [n=84]		Baseline Staff Population [n=88]		Staff Longitudinal Cohort [n=48]	
**Characteristics**	**Valid**	**n [%]**	**Valid**	**n [%]**	**Valid**	**n [%]**	**Valid**	**n [%]**	**Valid**	**n [%]**
**Gender – Male**	227	190 [83.7]	139	139 [100]	83	83 [100.0]	88	51 [58.0]	48	32 [66.7]
**Age in Years – Mean, SD**	224	40.2 [10.2]	139	36.7 [9.9]	82	37.2 [10.0]	85	45.8 [7.8]	45	48.0 [5.3]
**Highest level of education completed**			141		84					
Primary school or less				36 [25.8]		13 [15.5]				
Junior Cert				47 [33.3]		28 [33.3]				
Leaving Cert				28 [19.9]		16 [19.0]				
Diploma or Certificate				14 [9.9]		12 [14.3]				
Degree or higher				16 [11.3]		15 [17.9]				
**Months in Prison on this occasion**										
Median [IQR]			141	3 [5]	84	5.4 [4.8]				
**First time in prison**			140							
Yes				72 [51.4]	83	48 [57.8]				
**Regular employment prior to prison**			140							
Yes				69 [49.3]	83	46 [55.4]				
**Accommodation prior to prison**			141		84					
Homeless				7 [5.0]		3 [3.6]				
Living with parents				37 [26.4]		28 [33.3]				
Rented accommodation				76 [54.3]		39 [46.4]				
Own Home				20 [14.3]		14 [16.7]				
**Smoking Characteristics**										
Current Smoker	227	108 [47.4]	140	90 [64.3]	84	51 [60.7]	87	18 [20.7]	47	7 [14.9]
Past Smoker		55 [24.2]		21 [15.0]		14 [16.7]		34 [39.1]		20 [42.6]
Never Smoker		64 [28.2]		29 [20.7]		19 [22.6]		35 [40.2]		20 [42.6]

Table S5. Differences Between Quitters and Continued Smokers in Prisoner Population.

	Quitters [n=23]		Continued Smokers [n=28]	
	**Valid**	**n [%]**	**Valid**	**n [%]**
**Fagerstrom Category**				
Low	22	5 [22.7]	24	4 [16.7]
Low to moderate		4 [18.2]		7 [29.2]
Moderate		9 [40.9]		12 [50.0]
High		4 [18.2]		1 [4.2]
**Smoking Cessation Programme:**	**Valid**	**n [%]**	**Valid**	**n [%]**
Participated	23	23 [100.0]	27	9 [33.3]
Completed	23	23 [100.0]	27	8 [29.6]
**During last attempt to give up:**				
Used nicotine patches*	22	22 [100.0]	14	11 [78.6]
Support group for prisoners	22	22 [100.0]	14	8 [57.1]

*or gum, lozenges or spray.
